# Learning a hierarchical representation of the yeast transcriptomic machinery using an autoencoder model

**DOI:** 10.1186/s12859-015-0852-1

**Published:** 2016-01-11

**Authors:** Lujia Chen, Chunhui Cai, Vicky Chen, Xinghua Lu

**Affiliations:** Department of Biomedical Informatics, University of Pittsburgh, 5607 Baum Blvd, 15237 Pittsburgh, PA USA

**Keywords:** Yeast, Transcription, Gene expression, Transcriptomic machinery, Signal transduction, Deep learning, Deep hierarchical neural network, Unsupervised learning, Data mining

## Abstract

**Background:**

A living cell has a complex, hierarchically organized signaling system that encodes and assimilates diverse environmental and intracellular signals, and it further transmits signals that control cellular responses, including a tightly controlled transcriptional program. An important and yet challenging task in systems biology is to reconstruct cellular signaling system in a data-driven manner. In this study, we investigate the utility of deep hierarchical neural networks in learning and representing the hierarchical organization of yeast transcriptomic machinery.

**Results:**

We have designed a sparse autoencoder model consisting of a layer of observed variables and four layers of hidden variables. We applied the model to over a thousand of yeast microarrays to learn the encoding system of yeast transcriptomic machinery. After model selection, we evaluated whether the trained models captured biologically sensible information. We show that the latent variables in the first hidden layer correctly captured the signals of yeast transcription factors (TFs), obtaining a close to one-to-one mapping between latent variables and TFs. We further show that genes regulated by latent variables at higher hidden layers are often involved in a common biological process, and the hierarchical relationships between latent variables conform to existing knowledge. Finally, we show that information captured by the latent variables provide more abstract and concise representations of each microarray, enabling the identification of better separated clusters in comparison to gene-based representation.

**Conclusions:**

Contemporary deep hierarchical latent variable models, such as the autoencoder, can be used to partially recover the organization of transcriptomic machinery.

## Background

A cell constantly responds to its changing environment and intracellular homeostasis. This is achieved by a signal transduction system that detects the signals, assimilates the information of diverse signals, and finally transmits its own signals to orchestra cellular responses. Many of such cellular responses involve tightly regulated transcriptomic activities, which can be measured by microarray or RNA-seq technology and used as readouts reflecting the state of the cellular signaling system.

Reverse engineering the signaling system controlling gene expression has been a focus area of bioinformatics and systems biology. However, this task is significantly hindered by the following difficulties: 1) a transcriptomic profile of a cell (with contemporary technology, often a population of cells) at a given time represents a convolution of all active signaling pathways regulating transcription in the cells, and 2) the states of the majority of these signaling pathway are not observed, making it a challenging task to infer which genes are regulated by a common signal pathway, and it is even more challenging to reveal the relationships among signaling pathways.

Different latent variable models, such as principle component analysis [1], independent component analysis [2], Bayesian vector quantizer model [3], network component analysis [4–6], and non-negative matrix factorization [5, 6] models have been applied to analyze transcriptomic data, with an aim to represent the states of latent pathways using latent variables. Despite the different strengths and limitations of these models, they share a common drawback: the latent variables in these models are assumed to be independent, i.e., the latent variables are organized in single “flat” layer without any connection among them; as such the models lack the capability of representing the hierarchical organization of cellular signaling system.

**Fig. 1:**
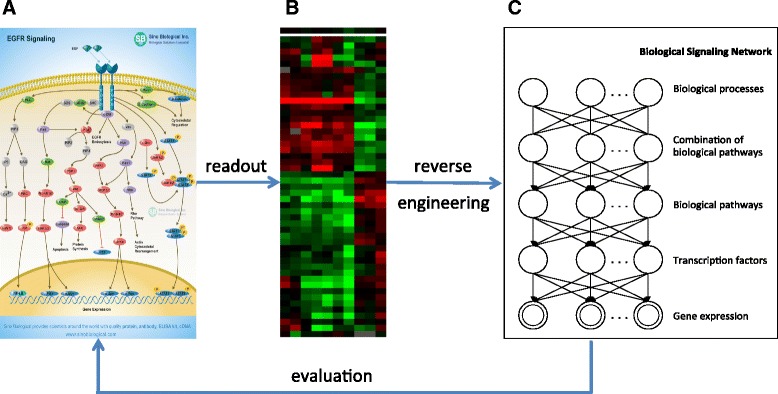
An overview of studying molecular signaling transduction using an autoencoder. **a** An example of molecular signaling transduction. **b** An example of the heatmap of gene expression microarrays. **c** An autoencoder model consisting of hierarchically organized hidden variables. After the model was trained, we evaluated the information learnt from the autoencoder model by testing whether the information carried by hidden variables in the autoencoder has real biological entities

Figure 1 illustrates the task of reverse-engineering a transcriptomic regulation system. Figure 1a illustrates the well-appreciated hierarchical organization of signaling molecules in cells and how the information encoded by signaling molecules are compositionally organized. It also shows that the convoluted signals eventually are emitted as changed gene expression. At this stage, all the hierarchical information of the signaling system is embedded in the data, a vector of gene expression value, in the form of context-specific and compositional covariance structures. When given a collection of transcriptomic profiles collected under different cellular conditions (Fig. 1b), the ultimate task is to recover the structure of the signaling systems shown in Fig. 1a, but the goal remains unattainable with current methodologies. In this study, we hypothesize that the hierarchical organization of cellular signals can be partially reconstructed by models capable of discovering and representing the context-specific and compositional covariance structure embedded in transcriptomic data. To this end, recent development in deep hierarchical models, commonly referred to as “deep learning” models, e.g., the autoencoder (deep belief network) shown in Fig. 1c, afford us the tools to reverse engineer the signaling systems of cells by mining systematic perturbation data.

In this family of deep hierarchical models, multiple layers of hidden (latent) variables are organized as a hierarchy, which can be used to capture the compositional relationships embedded in the transcriptomic data in a distributed fashion, i.e., different layers can capture different degrees of detail. For example, the relationships between TFs and their target genes can be captured by a hidden variable layer (hereafter referred to as hidden layer) immediately above the observed the layer of observed gene expression variables, whereas the function of pathways regulating TFs can be represented by higher hidden layers. Therefore, deep hierarchical models provide an abstract representation of the statistical structure of the transcriptomic data with flexibility and different degrees of granularity. We hypothesize that, if accurately trained, a deep hierarchical model can potentially represent the information of real biological entities and further reveal the relationships among them.

In this study, we designed and trained a sparse deep autoencoder model to learn how the information is encoded in yeast cells when subjected to diverse perturbations. Our results indicate that deep learning models can reveal biologically sensible information, thus learning a better representation of the transcriptomic machinery of yeast, and we believe that the approach is applicable to more complex organisms.

## Methods

In this study, we investigated using the autoencoder model [7] and sparse autoencoder model [8] to represent the encoding system of the signal transduction systems of yeast cells. Before introducing the autoencoder model and sparse autoencoder model, we will first briefly review restricted Boltzmann machines (RBMs) as building blocks for the autoencoder.

### Restricted Boltzmann Machines (RBMs)

A RBM is an undirected probabilistic graphical model that consists of two layers of stochastic binary variables (represented as nodes in the graph): a visible layer *v* ∈ {0, 1}^*D*^ and a hidden layer *h* ∈ {0, 1}^*F*^. The energy function *E* of the state {*v, h*} of the RBM is:

$$ \mathrm{E}\left(v,h;\theta \right)=-{a}^{\intercal }v-{b}^{\intercal }h-{v}^{\intercal } Wh = -{\displaystyle \sum_{\mathrm{i}=1}^{\mathrm{D}}}{\mathrm{a}}_{\mathrm{i}}{\mathrm{v}}_{\mathrm{i}}-{\displaystyle \sum_{\mathrm{j}=1}^{\mathrm{F}}}{\mathrm{b}}_{\mathrm{j}}{\mathrm{h}}_{\mathrm{j}}-{\displaystyle \sum_{\mathrm{i}=1}^{\mathrm{D}}}{\displaystyle \sum_{\mathrm{j}=1}^{\mathrm{F}}}{\mathrm{v}}_{\mathrm{i}}{\mathrm{h}}_{\mathrm{j}}{\mathrm{w}}_{\mathrm{i}\mathrm{j}} $$

In this equation, the binary state of visible variable i is represented by v_i_, the binary state of hidden variable j is by h_j_ and the model parameters are *θ* = {*a*, *b*, *W*}. The bias for visible variable i is a_i_ the bias for hidden variable j is b_j_ and the weight between visible variable i and hidden variable j is w_ij_.

The joint distribution of the hidden and visible variables is defined using a Boltzmann distribution, and the conditional probability of the states of hidden variables and visible variables are as follows:

$$ \begin{array}{c}\hfill Pr\left(v,h;\theta \right)=\frac{1}{Z\left(\theta \right)} \exp \left(-\mathrm{E}\left(v,h;\theta \right)\right)\hfill \\ {}\hfill Z\left(\theta \right)={\displaystyle \sum_{v,h}} \exp \left(-\mathrm{E}\left(v,h;\theta \right)\right)\hfill \\ {}\hfill Pr\left({\mathrm{h}}_{\mathrm{j}}=1\Big|v\right)=\sigma \left({\mathrm{b}}_{\mathrm{j}}+{\displaystyle \sum_{i=1}^m}{\mathrm{W}}_{\mathrm{i}\mathrm{j}}{\mathrm{v}}_{\mathrm{i}}\right)\hfill \\ {}\hfill Pr\left({\mathrm{v}}_{\mathrm{i}}=1\Big|h\right)=\sigma \left({\mathrm{a}}_{\mathrm{i}}+{\displaystyle \sum_{j=1}^n}{\mathrm{W}}_{\mathrm{i}\mathrm{j}}{\mathrm{h}}_{\mathrm{j}}\right)\hfill \end{array} $$

where σ(x) is the logistic function 1/(1 + exp(−x)), m is the total number of visible variables and n is the total number of hidden variables.

The efficient algorithm for learning parameters of the RBM model was introduced in detail in literature and our previous work [7, 9, 10].

### Autoencoder

Unlike a RBM, which captures the statistical structure of data using a single layer of hidden nodes, an autoencoder uses multiple layers in a distributed manner, such that each layer captures the structure of different degrees of abstraction. As shown in Fig. 1c, an autoencoder contains one visible (input) layer and one or more hidden layers. To efficiently train the autoencoder, we treat it as a series of two-layered restricted Boltzmann machines (RBM) stacked on top of each other [7, 9]. The inference of the hidden node states and learning of model parameters are performed by learning the RBM stacks bottom-up, which is followed by a global optimization of generative parameters using the back-propagation algorithm. More details of the algorithm and pseudo code for training an autoencoder were discussed in both literature and our previous work [7, 9, 10].

### Sparse autoencoder

In a conventional RBM model, each hidden unit is fully connected to the observed variables. After training, there is usually a non-zero weight between each pair of visible and hidden nodes. Based on the assumption that the change in gene expression due to a specific perturbation—most microarrays in this study are experiment-vs-control—is likely meditated by a small number of TFs or pathways, we adopted the sparse autoencoder model [8, 11] to simulate the cellular response to perturbations. The sparse autoencoder model enables one to specify that only a certain percent of hidden nodes have a high probability to be set to 1 (“on”) by adding a penalization term to the optimization function. Optimization of the traditional RBM is performed by minimizing the negative log-likelihood of the data during RBM training within an autoencoder:

$$ {\mathrm{minimize}}_{\left\{\theta \right\}}-{\displaystyle {\sum}_{1=1}^{\mathrm{s}} \log }{\displaystyle {\sum}_{\mathrm{j}=1}^{\mathrm{n}} Pr\left({\mathrm{v}}^1,\kern0.5em {\mathrm{h}}_{\mathrm{j}}^1\left|\theta \right.\right)} $$

Where s is the total number of samples, n is the total number of hidden units and *θ* = {*a*, *b*, *W*}. The sparse RBM adds the regularization term [8] into the optimization:

$$ {\mathrm{minimize}}_{\left\{\theta \right\}}-{\displaystyle \sum_{\mathrm{l}=1}^{\mathrm{s}}} \log {\displaystyle \sum_{\mathrm{j}=1}^{\mathrm{n}}} Pr\left({\mathrm{v}}^{\mathrm{l}},{\mathrm{h}}_{\mathrm{j}}^{\mathrm{l}}\Big|\theta \right) + \uplambda {\displaystyle \sum_{\mathrm{j}=1}^{\mathrm{n}}}\left|\mathrm{p}-\frac{1}{\mathrm{s}}{\displaystyle \sum_{\mathrm{l}=1}^{\mathrm{s}}}\mathrm{E}\left[{\mathrm{h}}_{\mathrm{j}}^{\mathrm{l}}\Big|{\mathrm{v}}^{\mathrm{l}}\right]\right|{}^2 $$

Where *λ* is the regularization constant and p is a constant (usually representing the percent of nodes desired to be on) controlling the sparseness of the hidden units h_j_. For the traditional RBM, the parameters are updated just based on the gradient of the log-likelihood term. But for the sparse RBM, the parameters are updated not only based on the gradient of the log-likelihood term but also the gradient of the regularization term.

### Non-negative matrix factorization

Non-negative matrix factorization (NMF) has been applied to reduce the dimension of expression data from thousands of genes to a handful of hidden representations (ex. metagenes) [5]. NMF is an algorithm based on decomposition by parts that can reduce the dimension of a matrix V [6].

$$ \mathrm{V}=\mathrm{W}\ast \mathrm{H} $$

Given that the gene expression data is represented as matrix V, NMF factorizes it into a basis matrix (W) and a coefficient matrix (H). All three matrices should have non-negative elements. The number of hidden regulators is pre-defined, and is usually much smaller then the number of genes. In this study, we used the Matlab function nonnegative matrix factorization “nnmf” to perform NMF analysis.

### Model selection of autoencoder and sparse autoencoder

We performed a series of cross-validation experiments to search for an “optimal” structure for autoencoders and sparse autoencoders. We adopted a four-layered autoencoder to represent the hierarchical structure of biological processes shown in Fig. 1c. We then explored models with different numbers of hidden units in each hidden layer. We set the initial structure of both autoencoder and sparse autoencoder to the following ranges: h^(1)^: 100–428; h^(2)^: 50–100; h^(3)^: 50; and h^(4)^: 25. We iteratively modified the structure of the model by changing the number of hidden nodes within a layer using a step size of 50 for the first and second hidden layer. Then we explored all combinations in the range stated above. In this case, the total number of models tested is 14 (7*2) for both autoencoder and sparse autoencoder. For the sparse autoencoder, we chose three sparsity constants that are 0.05, 0.1 and 0.15. Under each particular setting, we performed ten fold cross-validation to assess the performance of a model.

We used two criteria of evaluating the performance of the models. One is the reconstruction error, which is the difference between the original input data and the reconstructed data after training the model [12]. Due to the sparse features of the sparse autoencoder, we used Bayesian information criterion (BIC) [13] as another criteria for comparing models. BIC combines the factors of likelihood and number of free parameters to be estimated. The model with the lowest BIC is preferred.

$$ \begin{array}{l}\mathrm{B}\mathrm{I}\mathrm{C} = -2\cdot \ln \widehat{\mathrm{L}}+\mathrm{k}\cdot \ln \left(\mathrm{n}\right)\\ {}\widehat{\mathrm{L}}={\displaystyle \prod_{\mathrm{i}=1}^{\mathrm{N}}{\mathrm{p}}^{\mathrm{m}}}{\left(1-\mathrm{p}\right)}^{1-\mathrm{m}}\end{array} $$

where $\widehat{\mathrm{L}}$ is the maximized value of the likelihood function of the model, k is the number of free parameters to be estimated, n is the number of samples, p is the probability predicted from the model for a gene to be active in an experiment, and m is the true binary state of a unit in the input data.

### Mapping between the hidden units and known biological components

Based on the weights between each hidden unit in the first hidden layer and all the visible units (genes), we used a threshold (top 15 % of the absolute values of weights) to cut the edges between a hidden node and the observed genes, such that an edge indicates that the hidden node regulates the gene. We then identified all genes predicted to be regulated by a hidden node as a gene set. Based on the DNA-Protein interaction table [14, 15], we also identified the gene set regulated by a known TF. We then assessed the significance of overlapping of gene sets regulated by hidden nodes and TFs using hypergeometric testing.

### Consensus clustering of experiment samples

Consensus cluster clustering [16] was used to cluster the experiment samples using different datasets as input. The R implementation of ClusterCons [17] was downloaded from CRAN (https://cran.r-project.org/src/contrib/Archive/clusterCons/). The inputs for consensus clustering are the samples represented using original gene expression values, NMF megagenes values and the states of hidden variables under all experiment samples respectively. The partition around medoids (PAM) and *K*-means algorithms were used as base clustering algorithms. The inputs for cluster by cluster consensus clustering are the samples represented using samples clusters derived from the nodes from different hidden layers as features. If one sample belongs to a sample cluster, its input value is 1. Otherwise, its input value is 0.

### Finding pheromone related hidden units

We calculated the significance between the state of a hidden node and the state of proteins related to pheromone signaling pathway by using the chi-square test. First, we used a threshold (top 15 %) to designate the state of a hidden unit as active or inactive based on its activation probability. Then, for each hidden unit, we created a contingency table to collect the counts of the joint state of the hidden node and whether any member of the pheromone pathway is perturbed in a specific experiment. We used the contingency table to perform the chi-square test. We used a *p*-value of 0.01 as the significance threshold.

### Gene ontology analysis

GO [18] provides a standard description of what gene products do and where they are located. One of the frequently used databases that provide GO information for yeast is Saccharomyces Genome Database SGD. We first used the combination of weights [19] between neighboring hidden layers to get the weights between the hidden units in a particular hidden layer and the genes. A gene is regarded as being regulated by a hidden unit if their weight is in the top 15 % of all weights. When a gene set of interest associated with a hidden unit is available, we used the method mentioned in [20] to summarize the GO terms capturing as much as semantic information associated with those genes [21]. We identified the GO terms that could summarize the largest number of genes, while undergoing a minimal information loss.

## Results and discussion

### Training different models for representing yeast transcriptomic machinery

We collected a compendium of 1609 yeast cDNA microarrays from the Princeton University Microarray Database (puma.princeton.edu), and we combined them with 300 microarrays from the study by Hughes et al. [22], which was used in a previous study of the yeast signaling system [23]. The combined dataset is ideal for studying the yeast signaling system because it represents a large collection of perturbation experiments that are of biological interest. For example, the data from the study by Hughes et al. [22] were collected from yeast cells with genetic perturbations (deletion of genes) or chemical treatments, and similarly the microarrays from the database were collected from specific conditions and contrasted with “normal” growth condition. Taking advantage of the experiment-vs-control design of cDNA microarrays, we identified differentially expressed genes (3-fold change) in each array and retained 2228 genes that were changed in at least 5 % of the microarrays. We then represented the results of each microarray experiment as a binary vector, in which an element represented a gene, and its value was set to 1 if the gene was differentially expressed, and 0 otherwise. Thus, each microarray represented the *transcriptomic changes* in response to a certain condition, presumably regulated by certain specific signaling components, which is unknown to us.

We investigated the utility of the autoencoder model (also known as deep belief network) [9], with one observed layer representing the microarray results and 4 hidden variable layers (hereafter referred to as hidden layers) representing the yeast signaling components in yeast transcriptomic machinery. In this model, a hidden node is a binary variable, which may reflect the state of a collection of signaling molecules or a pathway, such that the switching of the node state between 1 and 0 can reflect the changing state of a pathway.

The probabilistic distribution of the state of a node in a given layer is dependent on the nodes in the adjacent parent layers, defined by a logistic function. The directed edges between nodes of adjacent layers indicate that, conditioning on the state of nodes in parent layer, the nodes in a child layer are independent. In other words, the statistical structure (patterns of joint probability of nodes) among the nodes in a child layer is captured by the nodes in the parent layer. For example, in our case, if the nodes in the 1^st^ hidden layer (directly above the gene expression layer) represent the states of transcription factors, then co-differential expression (covariance) of a set of genes is solely dependent on (or explained by) the TFs that regulate the genes. Similarly, the co-regulation of TFs is determined by its parent layer, which may reflect the state of signaling pathways. Thus, this model is suited to capture the context-specific changes and compositional relationship among signaling components in a distributed manner. The model is referred to as autoencoder because, when given a collection of observed data, it learns to use hidden nodes to encode the statistical structure of observed data, and it is capable of probabilistically reconstructing the observed data.

Since the autoencoder model in our study is biologically motivated, we hypothesize that the nodes in the first hidden layer would likely capture the signal of TFs. Thus the number of nodes in this layer should be close to the number of known TFs for yeast, of which there are around 200 well-characterized yeast TFs [24]. However, for a given microarray from a perturbation experiment, genes that respond to a specific perturbation are likely regulated by a few transcription factors. Thus we also investigated a model referred to as *sparse autoencoder* [8, 25], which performs regularized learning of model parameters and allows a user to constrain the percent of nodes in a layer that can be set to the “on” state, see Methods for details. In our experiment, we constrained that, in the first hidden layer, around 10 % of hidden nodes should be used to encode the changes in a microarray.

**Fig. 2:**
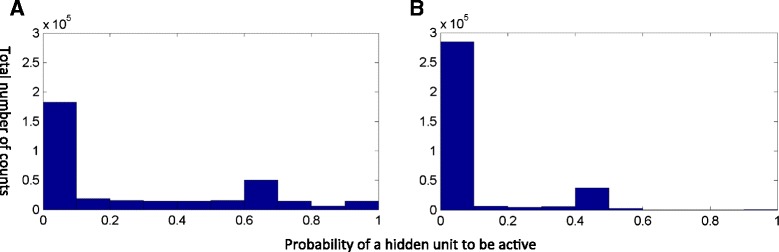
The histogram of the expected states of hidden units (probability of hidden units to be on) in the first hidden layer for the conventional autoencoder (**a**) and sparse autoencoder (**b**) respectively. For both models, the number of hidden units from the first hidden layer to the fourth hidden layer is 214, 100, 50, and 25 respectively. The sparsity threshold for the sparse autoencoder is 0.1. A hidden unit has a state under each experiment condition. Therefore, the total number of states for all hidden units is the number of experiment condition (1609) * the number of hidden units (214). The x-axis is the probability of a hidden unit to be on ranging from 0 to 1. The y-axis is the count of states

We first evaluated how adding a sparse regularization term influenced the state of hidden units (the probability of hidden units to be active/on). We trained a conventional autoencoder and a sparse autoencoder (setting the sparsity constraint to 10 %) using the microarrays. For each microarray, the models probabilistically inferred the state of each hidden node (the probability of a node to take a value of 1). Figure 2 shows the histogram of the expected states of the nodes in the first hidden layer associated with all microarray samples. In the conventional autoencoder, a relatively larger number of the nodes in the first hidden layer had a non-zero probability to be 1 (“on state”) (Fig. 2a), whereas the majority of the hidden nodes in the sparse autoencoder model were expected to take a value of 0 (“off state”) (Fig. 2b). Thus, the sparse autoencoder strives to use less hidden nodes to encode the same statistical structure in the observed data, instead of using every hidden node, with each contributing a little to the expression of genes. This is a desired property conforming to our assumption that the response to a specific perturbation should be encoded by a relatively small number of TFs.

We further evaluated how well models with different architectures (mainly concentrating on the number of hidden nodes in the first hidden layer) can be used to represent the transcriptomic machinery of yeast. Table 1 shows the results of a limited model selection experiment based on our biological assumptions (note that an exhaustive search of possible combinations of architecture and parameter settings is intractable). We calculated the reconstruction error, which is the sum of the differences across all microarrays between the observed expression state (0 or 1) of genes in microarrays and the expected states of genes reconstructed by the autoencoder. The results indicate that models of the same type (conventional or sparse autoencoder) could learn to encode data with a similar accuracy across the range of the architectures studied here, although the sparse autoencoder had higher reconstruction errors.

**Table 1:** Reconstruction error of models with different architectures

Reconstruction error	Architecture 1 (100:100:50:25)	Architecture 2 (150:100:50:25)	Architecture 3 (214:100:50:25)	Architecture 4 (428:100:50:25)
Autoencoder training	150.20	150.23	148.94	150.81
Autoencoder test	188.57	189.12	190.76	189.63
Sparse autoencoder training (0.1)	170.19	170.07	170.41	171.94
Sparse autoencoder test (0.1)	206.58	208.40	203.99	203.27

While the results indicate that the reconstruction errors of sparse autoencoder models were a bit higher than the ones of the traditional autoencoder, it should be noted that the sparse autoencoder reconstructed the same data with a much smaller number of hidden variables. From the perspective of the minimum description length (MDL) principle [26], a model is preferred if it can encode the information of a dataset with a minimal description length while achieving a similar or better reconstruction of data. In information theory, the description length is measured as the number of bits needed to encode the data, and in our case each bit is encoded by a hidden node. Thus, the sparse autoencoder potentially is a more desirable model even if it suffers a higher reconstruction error. To quantify and compare the utility of conventional and sparse autoencoders, we calculated the Bayesian information criteria (BIC) of the models, and the results are shown in Table 2. The results indicate that the BIC of the sparse autoencoder with an architecture consisting of hidden layers with 214, 100, 50, 25 hidden nodes (1^st^ to 4^th^) respectively is the lowest (the best) among the compared models. Since the number of hidden nodes in the first hidden layer of this model agrees better with the knowledge of the number of transcription factors, we chose to investigate the results derived from this model in the following sections.

**Table 2:** BIC scores of different models

	**Arch 1** (100,100,50,25)	**Arch 2** (214, 100, 50, 25)
**Autoencoder**	**3.25e + 006** = 1.99e + 006 + 1.26e + 006;	**4.41e + 006** = 1.71e + 006 + 2.70e + 006;
**Sparse autoencoder**	**2.06e + 006** = 1.93e + 006 + 1.26e + 005;	**1.96e + 006** = 1.69e + 006 + 2.70e + 005;

The cells show the BIC score (bold) and the individual terms of the BIC (see Methods). The numbers in the parentheses associated with each architecture (Arch) indicate the number of hidden nodes in 1^st^ – 4^th^ hidden layers

### Distributed representation enhances discovery of signals of TFs

**Fig. 3:**
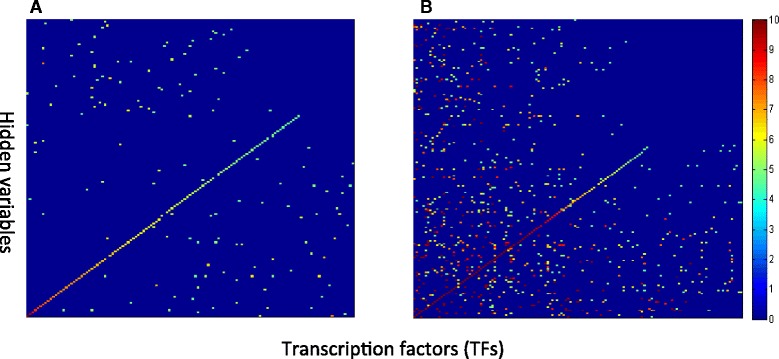
Mapping between transcription factors (TFs) and hidden variables in the first hidden layer. Results for sparse autoencoder (**a**) and NMF (**b**) are shown. The transcription factors (TFs) are arranged along x-axis, and the hidden variables are arranged along y-axis. Each point in the figure represents the value of –log(*p-value*) of the enrichment score between genes regulated by a hidden node and a TF. The pseudo-color bar shows the scale of the –log(*p-value*)

The motivation of using a hierarchical model is to allow latent variables in different hidden layers to capture information with different degree of abstraction in a distributed manner. When modeling transcriptomic data, one goal is to discover the signals of TFs. In a sparse autoencoder, it is natural to expect that the 1^st^ hidden layer should capture the signals encoded by TFs. We test this hypothesis by evaluating the overlap of the genes predicted to be regulated by a hidden node in the 1^st^ hidden layer and those known to be regulated by a TF. A statistically significant overlapping between them is shown in Fig. 3.

Indeed, the results indicate that sparse autoencoder is capable of capturing and representing the information of TFs, in that there is an almost one-to-one mapping between hidden nodes and known TFs as shown in Fig. 3a. For a few hidden variables that are significantly mapped to multiple TFs, we further investigated if these TFs are members of known TF complexes [27]. As an example, we found that a hidden node is significantly mapped to *AFT1* and *PUT3*, which are two yeast TFs known to cooperatively regulate genes involved in ion transportation [28]. As another example, a hidden node is mapped to both *MSN2* and *MSN4* [29], which belong to the same family and form hetero-dimers to regulate genes involved in general stress response.

To demonstrate the advantage of hierarchical and distributed representations, we compare our results with another latent variable model commonly used to represent microarray data, the non-negative matrix factorization model (NMF) [6]. The NMF can be thought of as a model consisting of an observed layer (gene expression) and a single hidden layer (hidden variables/metagenes [5]), which is used to capture all signals in embedded in microarrays, whereas the same information is distributed to multiple layers of hidden variables in the sparse autoencoder. We trained a NMF model with 214 “metagenes”, which is the same as the number of hidden nodes in the 1^st^ hidden layer of the sparse autoencoder, and the results of mapping between latent factors and TFs are shown in Fig. 3b.

Indeed, the results clearly demonstrate the expected difference between the two models. With the capability of capturing the context-specific and compositional relationship of signals regulating expression in a distributed manner, the hidden nodes in the 1^st^ hidden layer clearly capture the specific signals of TFs, whereas the signals regulated TFs are delegated to the higher level hidden nodes. In contrast, with only a single layer of latent variables, all signals in the data need to be “squeezed” into these latent variables, such that a latent factor (a “metagene”) has to represent the signal of multiple TFs. Therefore, the results support our hypothesis that, benefitting from the distributed representation of the statistical structures across multiple hidden layers, the sparse encoder can concisely learn and represent the information of biological entities, in this case the TFs.

### Latent variables can capture the information of signaling pathways

We further investigated whether certain hidden nodes can represent the states of well-known yeast signaling pathways, i.e., whether the state of a hidden node can be mapped to the state of a collection of proteins in a pathway. In a previous study [23], we were able to recover the pheromone signaling pathway and a set of target genes whose transcription were regulated by the pheromone pathway, by mining the systematic perturbation data from the study by Hughes et al. [22] in which 14 genes involved in yeast pheromone signaling were perturbed by gene deletion. In the current study, we identified the microarray experiments in which the aforementioned 14 pheromone-pathway genes were perturbed, and we examined if the state of any hidden node was statistically associated with perturbation of pheromone pathway, using the chi-square test (see Methods). Interestingly, we found 2 hidden nodes in the 1^st^ hidden layer that are significantly associated with the perturbation experiments, one with a chi-square test *p* ≤ 2.47e-05, and the other with a *p* ≤ 3.82e-02. Further inspecting the genes predicted to be regulated by these hidden nodes, we found a significant overlap between the pheromone target genes from our previous study and the genes regulated by these hidden nodes (data not shown). These results indicate that the hidden nodes of the sparse autoencoder model are capable of capturing the signals of specific yeast pathways. However, it should be noted that, by design, a hidden node in the high level layers of the sparse autoencoder might encode the signals of multiple pathways that share strong covariance.

### The hierarchical structure captures signals of different degrees of abstraction

**Fig. 4:**
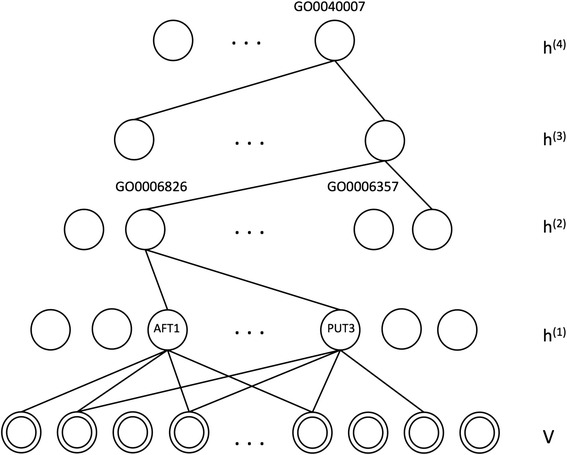
Example of hierarchical Gene Ontology (GO) map for hierarchical hidden structure

One advantage of the hierarchical structure of an autoencoder is to represent information with different degrees of abstraction. Intuitively, the lower level hidden layers should capture the highly specific signaling pathways or signaling molecules, such as TFs, whereas the high level hidden layers may encode more general information. To test this hypothesis, we identified the genes regulated by each hidden node by performing a linear weight combination experiment [19] (multiplication of weights between different hidden layers). We then applied a semantic analysis method previously developed by our group [20], which identifies the most appropriate concept from the Gene Ontology (GO) to summarize the genes. Interestingly, we found that genes regulated by AFT1 and PUT3 are significantly enriched among the genes regulated by a hidden node in the 2^nd^ hidden layer, and the genes regulated by this hidden node is summarized by the GO term GO:0006826 (*iron ion transportation*), shown in Fig. 4. Using the same method, we found another hidden node whose related genes were annotated with GO:0006357 (*regulation of transcription from RNA polymerase II promoter*). Thus, the results indicate that the distributed representation of information enables the hidden nodes at the different level of hierarchy to capture information of different degrees of abstraction.

### Concise representation enhances the discovery of global patterns

**Fig. 5:**
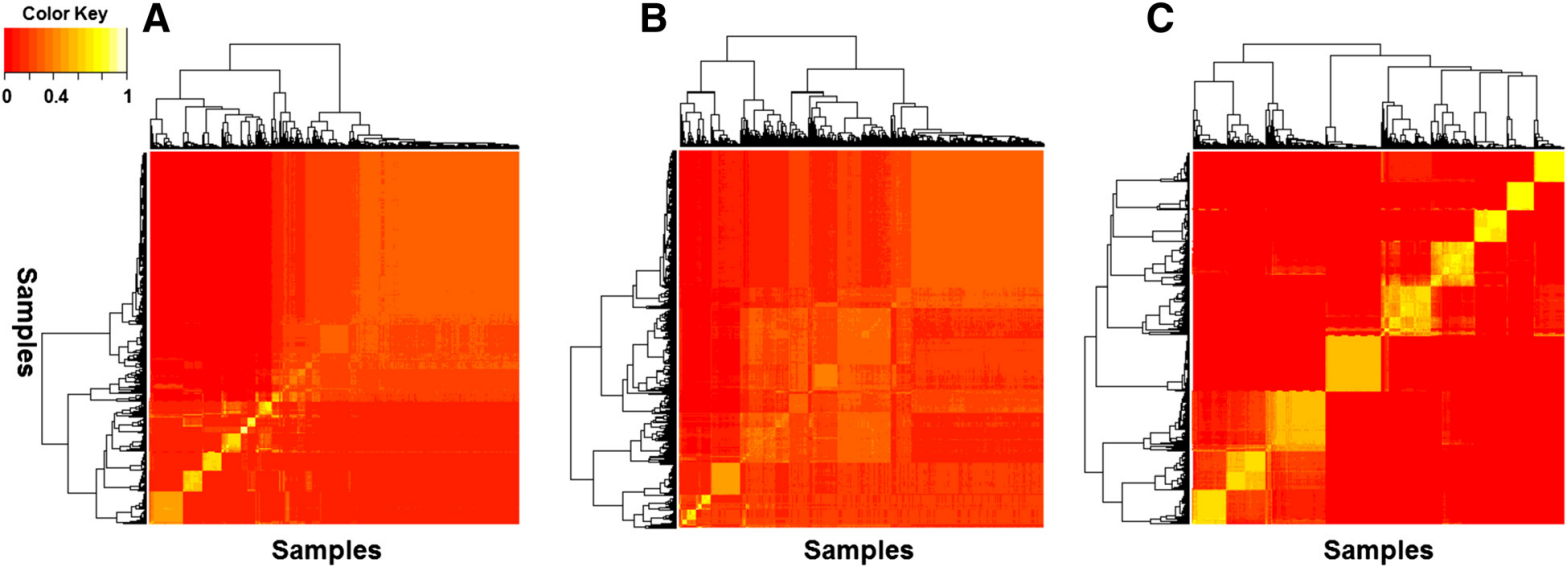
Clustering of experiment samples represented using original gene expression data (**a**), NMF metagenes (**b**) and expected state of hidden nodes in the first hidden layer (**c**). Consensus clustering results show how consistently a set of samples is assigned into a common cluster during repeated clustering experiments using samples with replacement from a dataset. A yellow box indicates a set of samples that are consistently assigned to a common cluster, and the brightness of yellow reflects the consistency

Given a large comprehensive dataset, it is often interesting to learn if distinct perturbations affect common biological processes of the cell [22, 23]. A common approach to discover such patterns is to perform clustering analysis and examine if certain samples (thereby experimental perturbations) are clustered together. In general, the result of a clustering analysis is significantly influenced by whether the features representing each sample are informative. Non-informative features may not reveal any real information, whereas features concisely reflecting the states of cellular signaling system may provide insights regarding the system. To examine if the signals represented by the latent variables are more informative than original gene expression values and NMF metagene values, we represented the samples in our dataset using the original gene expression values, NMF metagene values and the expected states of the hidden nodes in a hidden layer respectively, and we then compared the results of consensus clustering (Fig. 5).

The results clearly demonstrate that, if samples are represented in the high-dimensional gene expression space, the majority of the samples cannot be grouped into distinct clusters. When we use the low-dimensional NMF metagenes space, it performs slightly better than using original gene expression space. But, the clusters derived are still not well separated. In contrast, when samples were represented using the expected states of hidden nodes of the 1^st^ hidden layer, the samples can be consistently separated into distinct clusters. Representing samples using the expected states of the nodes from other hidden layers also produced clearly separated clusters (data not shown). The results indicate that the states of hidden nodes are much more informative in terms of representing distinct characteristics of individual samples, thus enabling clean separation of samples by the clustering algorithms. Although it would be interesting to systematically inspect the common characteristics of the samples in terms of whether the perturbation experiments affect common signaling pathways, such an analysis requires broad and in depth knowledge of yeast genetics, which is beyond the expertise of our group.

### Information embedded in data is consistently represented in different hidden layers

**Fig. 6:**
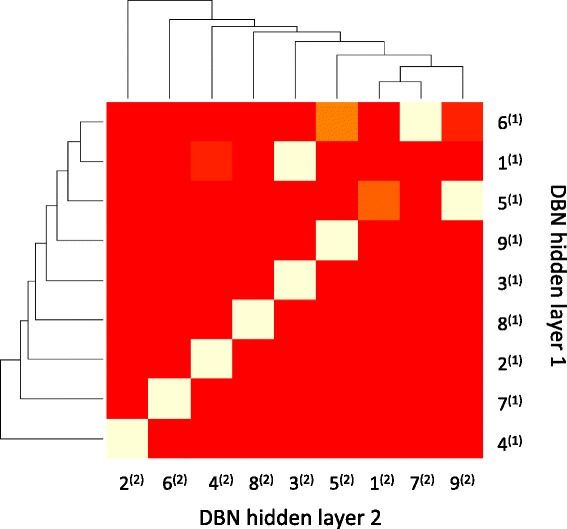
Cluster by cluster clustering for clusters in the 1^st^ hidden layer and the 2^nd^ hidden layer. The x-axis is the 9 sample clusters using the states of hidden units in the 2^nd^ hidden layer indexed by the superscript (2). The y-axis is the 9 samples clusters using the state of hidden units in the 1^st^ hidden layer indexed by the superscript (1). For example, 1^(1)^ is the first sample cluster using the states of hidden units in the 1^st^ hidden layer and 1^(2)^ is the first sample cluster using the states of hidden units in the 2^nd^ hidden layer. A yellow box indicates that the members of two clusters (derived from different representation) significantly overlap, with the brightness of yellow reflecting the degree of overlap

We hypothesized that, in a successful hierarchical representation of a dataset, the information embedded in data should be consistently encoded by different hidden layers, even though two different layers are of different dimensionality and identity of the nodes are totally different. In other words, when a sample is represented by the state of the nodes from different hidden layers, the characteristics that distinguish this sample from others (or make it similar to others) should be retained despite being represented by the nodes from different layers. To test this hypothesis, we first performed consensus clustering using nodes from different hidden layers as features to get sample clusters, and then we compare if members within a cluster derived using one representation significantly overlap with the members from another cluster derived using a different representation. Figure 6 shows the results of assessing the overlaps of the samples clusters derived using the nodes from the 1^st^ and 2^nd^ hidden layers as features respectively. The results indicate that the majority of the clusters derived with different representations agree. Interestingly, a cluster derived from the 2^nd^ hidden layer as features subsumes (maps to) two clusters derived using the 1^st^ hidden layer as features, indicating that the 2^nd^ layer captures more general information. The results indicate that, even though the dimensionality and identity of features of each hidden layer are significantly different, the information encoded by the hidden nodes in a sparse autoencoder is preserved across different layers.

## Conclusion

In this study, we investigated the utility of contemporary deep hierarchical models to learn a distributed representation of statistical structures embedded in transcriptomic data. We show that such a model is capable of learning biologically sensible representations of the data and revealing novel insights regarding the machinery regulating gene expression. We anticipate that such a model can be used to model more complex systems, such as perturbed signaling systems in cancer cells, thus directly contributing to the understanding of disease mechanisms in translational medicine.
